# Field Investigation of the Dynamic Response of Culvert–Embankment–Culvert Transitions in a High-Speed Railway

**DOI:** 10.3390/ma16175832

**Published:** 2023-08-25

**Authors:** Ping Hu, Huo Liu, Yi-Zhi Tang, Yu-Liang Lin

**Affiliations:** 1 Department of Engineering Management, Hunan University of Finance and Economics, Changsha 410205, China; huping@hufe.edu.cn (P.H.);; 2 School of Civil Engineering, Central South University, Changsha 410075, China

**Keywords:** culvert, high-speed railway, transition section, dynamic response, construction parameters

## Abstract

The stiffnesses of embankments and culverts differ in the transition sections of high-speed railways (HSRs) due to their different supporting conditions. The dynamic irregularity caused by the different stiffnesses makes this transition area the weakest part of high-speed railways. Graded crushed stone combined with 5% cement is typically used to fill the subgrade in these transition areas. Thus, three different particle size ratios of crushed stone were matched and tested regarding the construction parameters to explore the most suitable materials to fill the roadbed in a transition section. Then, field dynamic tests were carried out on the culvert–embankment–culvert transition area where trains run at speeds of 5–360 km/h. A time-domain analysis of the test data was performed to obtain the laws of variation that cause the dynamic characteristics to change with the railway line and roadbed layer and the changes induced by a train’s running speed, operating direction, and axle weight. The results indicate that (i) it is feasible to fill transition section roadbeds with well-graded crushed stone combined with 5% cement with optimal water contents; (ii) extreme dynamic responses in some special sections are observed, suggesting the value of taking special measures at the transition section. For example, the sections 14.5 m and 30 m from the 679 culvert and the bed layer should be specially stabilized; (iii) the train’s axle load and driving direction show a great effect on corresponding sections and layers but present a small effect on the sections and layers nearby; and (iv) 260 km/h is a critical speed.

## 1. Introduction

High-speed railways (HSRs) are renowned worldwide for their convenience, safety, and comfort [1,2,3]. In China, the running mileage of railways has increased to 42,000 km, and their speed of operation is up to 350 km/h [4,5]. In many other countries, future plans for developing HSR networks have also been proposed [6,7,8,9,10,11,12].

The dynamic irregularity induced by different stiffness and differential settlement in transitional zones during the long-term service of high-speed railways has been considered by many scholars [13,14,15,16,17]. Investigations issued by the European Rail Research Institute [18,19,20] showed that it was essential to take some special engineering measures in transition zones, particularly for embankments at bridge, tunnel, or culvert transitions. Banverket [21] and the Federal Railroad Administration (FRA) in the USA [22] indicated that the stiffness of embankment–bridge transition sections can double in value. Even worse, maintenance frequency at transition zones may be up to five times higher, requiring double the cost compared to normal sections. Presently, many measures have been put forward by researchers to deal with transition zones. A promising proposed solution involves the use of flowable fill materials to fill bridge–embankment transition areas, which can help improve the displacement resistance of a track’s supporting layer [23]. Another solution is to set a triangle or inverted triangle in a transition zone and use graded crushed stone combined with 5% cement to minimize the variation in the subgrade stiffness of various structures along the railway. Consequently, it is important to investigate the properties of roadbed fillers and the dynamic properties of embankments in transition sections.

Analytic solutions, as a fundamental method, have been widely used to explore the dynamic characteristics of geotechnical structures [24,25,26,27,28], thus providing a theoretical base for subsequent research. For example, Sajjad et al. [29] provided a method to adjoin sections with different stiffnesses to design a railway transition. However, it is still difficult to calculate the entirety of a transition whose subgrade stiffness changes along the line. Thus, some numerical simulation models were established to overcome the deficiencies of the analytic solutions [16,30,31,32]. The complicated real conditions of the railroads were simulated in numerical methods, and the calculated results were verified to be consistent with the field test results to ensure the correctness of the simulation [33,34,35,36,37]. Field test studies have been found to be capable of revealing the real dynamic properties of transition sections [38]. Some field tests have been carried out with trains operating at low speeds, while others have simply been aimed at ordinary railways. However, the current literature does not widely discuss dynamic properties in transition sections in HSRs [39,40,41,42,43,44,45,46].

The Wuhan–Guangzhou high-speed railway in China contains over 1900 culverts, 500 abutment bridges, 110 tunnels, and more than 1900 embankment and road-cutting junctions. The total number of these four types of transition sections is nearly 7000, distributed over a 300 km subgrade. A transition section is present every 45 m along the railway line. When two transition sections are close enough to each other, the dynamic waves under the train become excited, reflected, and superposed, causing more complicated dynamic responses than a normal transition section. Thus, a culvert–embankment–culvert transition section is selected for study in this paper. The performance of crushed stone with 5% cement is studied, and the dynamic properties of the culvert–embankment–culvert transition are tested when a high-speed-train is running at speeds ranging from 5 km/h to 360 km/h along the Wuhan-Guangzhou HSR. Finally, the time domain and the relationship between the dynamic responses, the railway’s direction and depth, and the train’s running direction, axle weight, and speed are discussed in this paper. The objectives of the work presented in this paper are: (i) to study the performance of a roadbed filling in a transition zone; (ii) to investigate the sensitive positions of a transition zone under a train’s load; (iii) to explore the factors of the transition zone that influence its dynamic responses; and (iv) to obtain the time-domain characteristics of the transition zone.

## 2. Material Properties and Construction Process

According to the *Code for the Design of High-Speed Railways (TB10621-2014)* [47], 3–5% cement must be mixed with crushed stone when filling the trapezoidal part of transition sections. Thus, indoor tests were conducted on graded crushed stone mixed with 5% cement, including measurement of the EDTA (ethylene diamine tetraacetic acid) content present in the cement, an unconfined compressive test, and a compaction test. On-site comparative experiment tests were conducted on the rolling effect under the optimal gradation with 5% cement content.

Six layers (with layer thicknesses of 30 cm, 30 cm, 30 cm, 25 cm, 25 cm, and 25 cm) were compacted by a 20 T vibrating roller on site. The graded crushed stone in culvert transition sections should be constructed with the A and B groups filling in the normal section that it connects with at the same time, and its rolling surface should be filled and compacted synchronously and evenly to the same horizontal height as the normal section. For both sides of the culvert, the filling process should be carried out symmetrically and synchronously with adjacent embankments. The sections close to the horizontal structure should be rolled parallel to the horizontal structure by a large roller. The parts that cannot be rolled by a large roller should be compacted in layers by small vibrating compaction equipment, and the paving thickness of the filling should not exceed 20 cm. The compaction times were determined through testing.

### 2.1. Assignment of Construction Equipment

**Figure 1:**
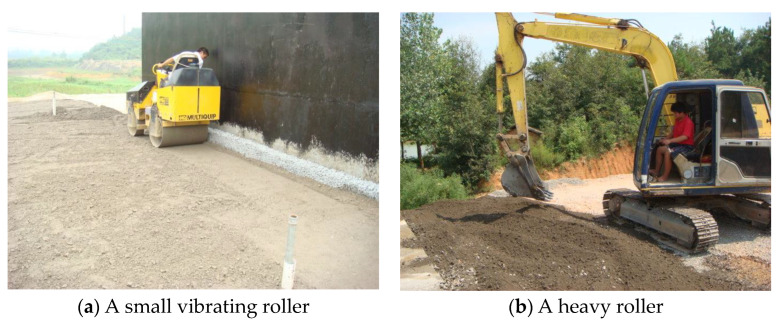
Some of the utilized construction machinery.

In terms of construction preparation, a variety of machinery and equipment were used for various processes, such as mixing, loading, transportation, leveling, and rolling machinery, in the construction process. The main construction equipment included a 750 lb site mixing system, an excavator, a bulldozer, a heavy roller, 2 dump trucks, a FL935E# loader, a grader, a paver, and other small-scale auxiliary equipment. Some of the equipment is displayed in Figure 1. Some conventional testing equipment was prepared for the monitoring tests, such as an EDTA dose titrator, heavy compaction equipment, K_30_ testing equipment, E_vd_ testing equipment, and E_v2_ testing equipment.

### 2.2. The Ratio of Graded Crushed Stone

Two proportions of mixing tests were conducted indoors to satisfy the gradation requirement according to the *Test Method for Railway Ballast (TB/T2328.14-2008)* [48]. At the same time, on-site filling and rolling process tests were conducted on the two graded crushed stone fillings to determine their optimal ratio. The first ratio was as follows: 5–31.5 mm crushed stone with a content of 35%, 5–25 mm crushed stone with a content of 30%, and 0–5 mm stone powder with a content of 35%. The second ratio was as follows: 5–31.5 mm crushed stone with a content of 30%, 5–16 mm crushed stone with a content of 20%, and 0–5 mm stone powder with a content of 50%.

After determining the gradation of graded crushed stone particles, it then became a key issue to determine their moisture content, as this determined whether the graded crushed stone could be compacted well. Based on a large amount of construction practice experience, it is easy to meet the compaction requirements when the moisture content of construction compaction is controlled at 4–6%.

### 2.3. Quality Inspection of Raw Filling Material

The raw materials used in the test section were tested according to the specification requirements for graded crushed stone. The testing items and results are revealed in Table 1; these materials were able to meet the specification requirements.

### 2.4. Grading of Gravel

**Figure 2:**
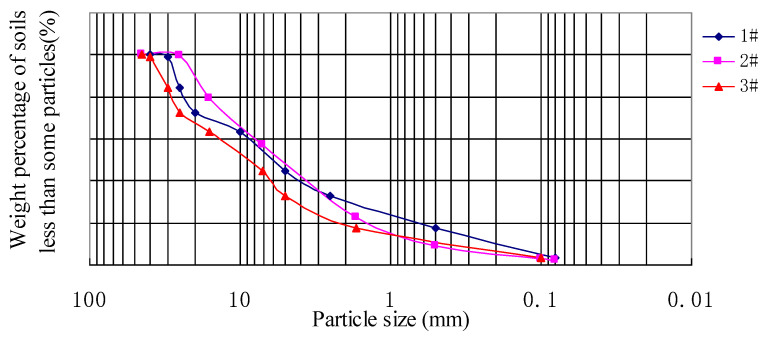
Grading curve of graded crushed stone.

The graded crushed stone that filled the culvert transition section of DK1252+679~+731 was mainly composed of sandstone. Three different particle size ratios of crushed stone were prepared to study its grading, compaction, and strength to determine the best grading ratio for filling the transition sections. The grading curves of these three graded crushed stones are displayed in Figure 2, and the grading indexes are shown in Table 2. We calculated that the uniformity coefficient of crushed stone #1 is $C_{u}=d_{60}/d_{10}=28.67$, while the values of the uniformity coefficient for crushed stone #2 and #3 are both 16. The curvature coefficient of crushed stone #1 is *C_c_*=1.53, and this value is 1.56 and 1.82, respectively, for crushed stones #2 and #3. The value of *C_u_* is larger than 10, and the value of *C_c_* is within a range of 1~3. It is seen that the grade distribution of crushed stone is not uniform; the crushed stone is well-graded and suitable for subgrade fillings. Furthermore, the grading parameters of mixed-grading crushed stone #1 are optimal.

### 2.5. Compaction Test and Unconfined Compressive Strength Test

The results of the graded crushed stone with 5% cement for the compaction test and for the test of its unconfined compression strength are shown in Table 3. The values of the unconfined compression strength of three samples are greater than 0.5 MPa, with a satisfactory strength to fill the transition subgrade.

### 2.6. Compaction or Rolling Effect

In the current codes, the strength index of the roadbed’s compaction quality adopts the foundation coefficients K_30_, E_v2_, E_vd_, and E_v2_/E_v1_ and a porosity value *n*. In Japan, scholars also adopt this indicator and determine the E_v2_ and E_vd_ indicators. In most countries in Europe, such as Germany, France, Austria, and Switzerland, E_v2_ and E_vd_ are adopted as strength indicators to control roadbed filling. In this study, K_30,_ E_v2_, E_vd_, E_v2_/E_v1_ and porosity *n* are adopted to evaluate the strength of the roadbed.

Through a comparison of our indoor test results, we can conclude that the unconfined compressive strength of crushed stone #2 is poorer than that of #1 and #3. Additionally, by comparing the results of #1 and #3, the optimal mix ratio can be determined according to three specifications. These tests were carried out on #2 stones with 6 repetitions of static pressing, and a comparison of these results is shown in Table 4.

Table 5 indicates that the K_30_ for #1 stones is larger than 190 MPa/m; similarly, its E_vd_ is greater than 55 MPa, E_v2_ is larger than 80 MPa, E_v2_/E_v1_ is less than 2.5, and its porosity is less than 28. Additionally, it satisfies the requirements of compaction set forth by the *Code for the Design of Subgrades of Railways (TB 10001-2016)* [49]. However, not all indicators of the #2 stones meet the specified requirements. It is seen that after compaction with cement, the #1 graded crushed stone has significant advantages regarding its tested indicators, such as its K_30_, E_v2_, E_v2_/E_v1_, and porosity. Therefore, its mixing ratio is the best among these three samples. The experiment shows that the better the grading is, the faster the ratio will decrease between E_v2_/E_v1_ and porosity *n*.

## 3. Field Test of the Transition Section

### 3.1. Brief Introduction of the Test Site

**Figure 3:**
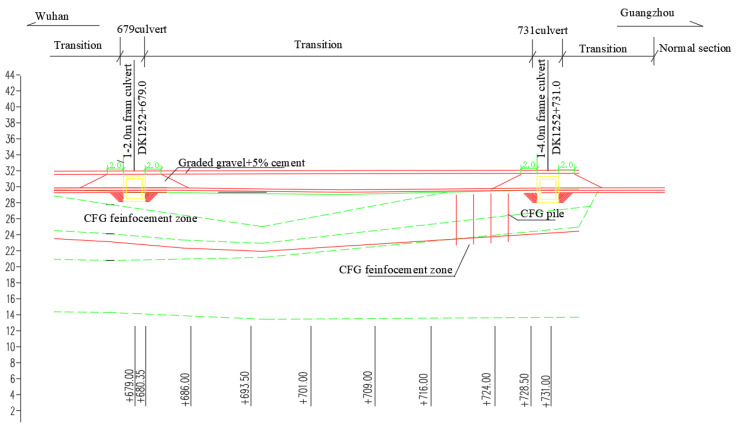
Longitudinal distribution of the culvert–embankment–culvert transition (unit: m).

The test section is a culvert–embankment–culvert transition in the Wuhan–Guangzhou HSR, starting from the mileage of DK1252 + 679 at the center of a frame culvert. The height of the culvert is 2.5 m, and its aperture is 2.0 m. The transition ends with the mileage of DK1252 + 731 at the center of another frame culvert. The height of the other culvert is 2.7 m, and its aperture is 2.0 m. The embankment is filled with a height of 1.7 m. The longitudinal distribution is shown in Figure 3.

### 3.2. Basic Test Parameters and Test Instruments

**Figure 4:**
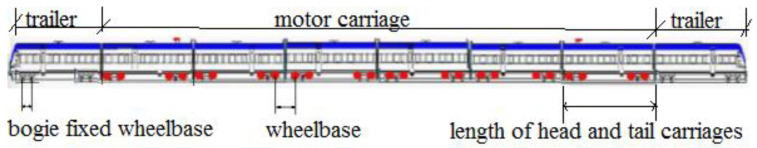
Diagram of a CRH2-type train.

**Basic test parameters:** The test train was a CRH2-068C-type train (see Figure 4) [50]. It had 8 carriages in total. The front and rear carriages were trailers whose length is 25,700 mm. There were 6 motor carriages between the front and the rear carriages, whose length was 25,000 mm. The axle load was less than 14 tons. The bogie wheel diameter was 860/790 mm, and the bogie fixed wheelbase was 2500 mm.

The running speed of the train was 5 km/h for the first test, the aim of which was to perform a quasi-static calibration at ground measurement points. Then, the speed of the train during the test gradually increased with the goal of ensuring safety, with the maximum tested speed reaching 360 km/h.

We use the term “downwards” to refer to train operations that run from north to south and use the term “upwards” to describe operations from south to north in this paper.

**Figure 5:**
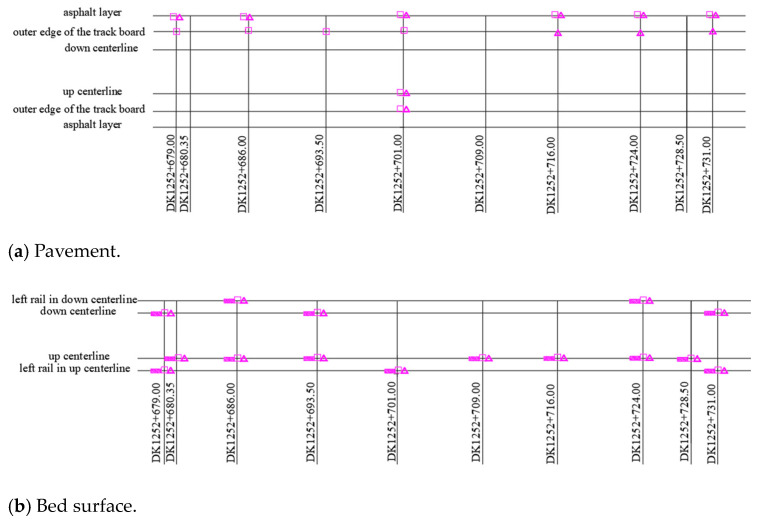

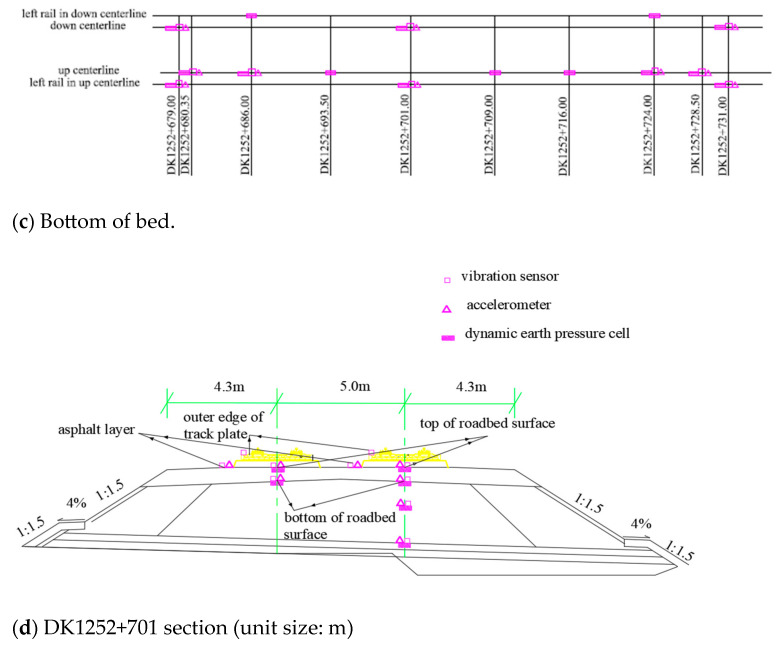
Distribution of test sensors (unit size: m). (Note: ■ represents a dynamic pressure cell, □ represents a vibration sensor, and △ represents an accelerometer).

**Test Instruments:** The test instruments included a signal collector, signal analysis software, and vibration sensors. The collector had 32 wireless channels and was able to collect 32 dynamic signals simultaneously. The signal analysis software was installed on a computer and performed operations such as simple integration, differentiation, and denoising on the data as soon as the signals were collected. Figure 5 shows the arrangement of the buried sensors, including the dynamic earth pressure cell, the accelerometer, and the vibration sensor.

All the instruments were calibrated before the tests, and all the cables were shielded through grouding before the tests.

**Buried position:** The test sensors were buried in 10 sections longitudinally along the railway: the middle of the culvert (DK1252 + 679 and DK1252 + 731), the side of the culvert (DK1252 + 680.35 and DK1252 + 728.5), the center of the transition section (DK1252 + 701), and the sections distributed among them (DK1252 + 686, DK1252 + 693.5, DK1252 + 709, DK1252 + 716, and DK1252 + 724).

The layers that were fitted with test sensors were, from the surface working downwards, the pavement, the bed surface, and the bottom of the bed. Many test sensors were embedded at the section DK1252 + 701 to investigate the dynamic behavior along the subgrade depth (see Figure 5c). It should be noted that the term “dynamic response” refers to the data collected from the bed surface layer in the following text, except for in the specialized study in the depth direction.

## 4. Signal Processing

The initial signals appeared to have burrs due to the presence of the noise. Thus, they were first filtered by the wavelet denoising method, in which a threshold form of VisuShrink and a soft threshold function were used to decompose the initial signals and form new signals. Then, the new signals were smoothed by the five-thirds smoothing method. Finally, all the data were tested by the K-test hypothesis of normal distributions.

It is worth noting that displacement cannot be directly measured. Thus, data on displacement were obtained by integrating the dynamic speed using the Newton–Cortege integral method, and they were proved accurate by comparing the integrated speed with the tested speed.

The processed data were analyzed using the mean square value, mean value, variance of random data signals, and the K test hypothesis of normal distributions. After analysis by the above method, any abnormal data that were 20% greater than the 95% confidence limit value were removed.

There were four types of dynamic measurement data in the transitional section: dynamic stress data, acceleration data, speed data, and dynamic displacement data.

The main process for analysis was as follows:

(1) A time domain analysis and statistical analysis were computed using the mean square, mean, and variance, a probability density function calculation, a joint probability density function analysis, and the Kolmogorov test.

(2) The variation of dynamic responses with the longitudinal, lateral, and depth positions was assessed.

The distribution characteristics of the dynamic response along the depth can be expressed as the decay percentage. For example, the dynamic stress σ can be expressed as

(1) $$m=(\sigma_{bs}-\sigma_{i})\times 100/\sigma_{bs}$$

where, *m* represents the percentage of dynamic stress attenuated from the bed surface to the subgrade layer, *σ_bs_* represents the dynamic stress on the bed surface, and *σ_i_* represents the dynamic stress on the *i^th^* subgrade layer.

(3) The dynamic response to the train’s speed and train’s axle load was assessed.

The impacts of axial weight on dynamic responses are indicated by the multiplication factor. For example, the impact on dynamic stress σ can be defined as

(2) $$n=(\sigma_{r}-\sigma_{d})/\sigma_{d}$$

where *n* represents the ratio of the difference in dynamic stress under the action of track inspection vehicles and high-speed trains to the dynamic stress under the action of high-speed trains; *σ_r_* represents the mean of the dynamic of the track inspection train at a certain depth; and *σ_d_* represents the mean of the dynamic of an HST at a certain depth.

In order to ensure the authenticity of the data, the test data underwent statistical analysis. Any data that were 20% greater or less than the 95% confidence interval were regarded as abnormal and were removed.

## 5. Dynamic Responses of the Transition Sections

All the data were tested by the above experiment and analyzed by signal analysis methods. To make the description more concise, “679 culvert” is an abbreviation of “the middle of the culvert in the DK1252 + 679 section”. In the same way, DK1252 + 701 is the section that is 22 m from the 679 culvert, DK1252 + 709 is the section that is 30 m from the 679 culvert, and so on. Additionally, “731 culvert” is an abbreviation of “the middle of the culvert in the DK1252 + 731 section”. “LDF6C” is short for “the longitudinal distance from culvert DK679 to the small mileage direction”; “DRABS” is short for “dynamic response amplitude on bed surface”. “DFBS” is short for “depth from bed surface”, “upward direction” means the direction from small mileage to big mileage, and “downward direction” means the direction from big mileage to small mileage. Finally, it should be noted that “response” means “stress”, “acceleration”, “speed”, and “displacement”.

### 5.1. Time–History Domain Analysis

**Figure 6:**
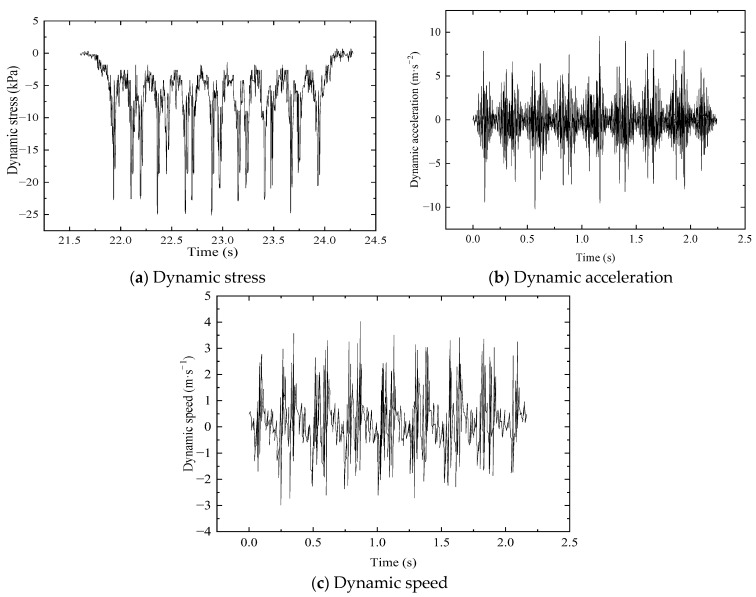
Time–history curves on bed surface at 679 culvert section.

The time–history curves on the bed surface at the 679 culvert section with a train speed of 350 km/h are displayed in Figure 6. This figure shows that dynamic responses become increasingly large when the wheels pass over the test section, while the dynamic response becomes increasingly smaller when the wheels pass away from the test section. This infers that the peaks and the troughs correspond to the time when the wheels pass over and away from the 679 culvert sections. Thus, a bogie passing is equivalent to two cycles of loading and unloading.

### 5.2. Responses along the Railway Line

**Figure 7:**
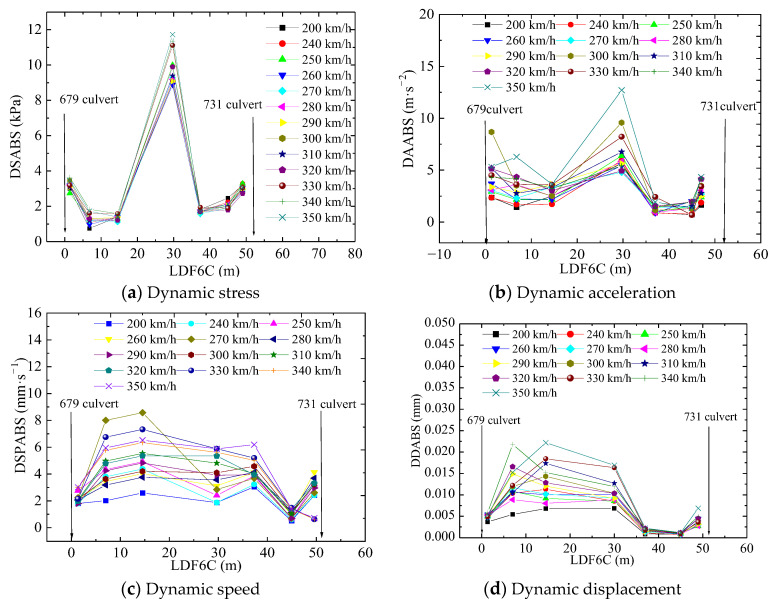
Changes in dynamic responses with longitudinal position when the train runs through the culvert–embankment–culvert transition.

Figure 7 shows changes in dynamic response along the railway line longitudinally at different train speeds. The curve first decreases to its minimum value for the section that is 6.8 m away from the 679 culvert and then increases to a peak value (see Figure 7a). A similar phenomenon is observed at the 731 culvert. This can primarily be attributed to the variation in the infill materials along the longitudinal line and the different layers along the transitional section. When a train runs through the transitional section, vibration waves are generated and transmitted throughout the roadbed. The waves subsequently refract and reflect when encountering different infill media, and thereby, the vibration waves are superposed again and again as they propagate throughout the whole transitional section. This results in the peak value that is observed at the section that is 29.7 m away from the 679 culvert.

The dynamic acceleration amplitude at the top of the two culverts is the most significant during train operations, and it decreases quickly at the side of the culvert (see Figure 7b). The peak acceleration is observed 29.7 m from the 679 culvert.

The maximum value of dynamic speed occurs 14.5 m and 37 m away from the 679 culvert, while the dynamic displacement reaches its maximum value 15 m or 30 m away from the 679 culvert (see Figure 7c,d), by which we can infer that the stiffness changes considerably.

### 5.3. Response along the Depth

**Figure 8:**
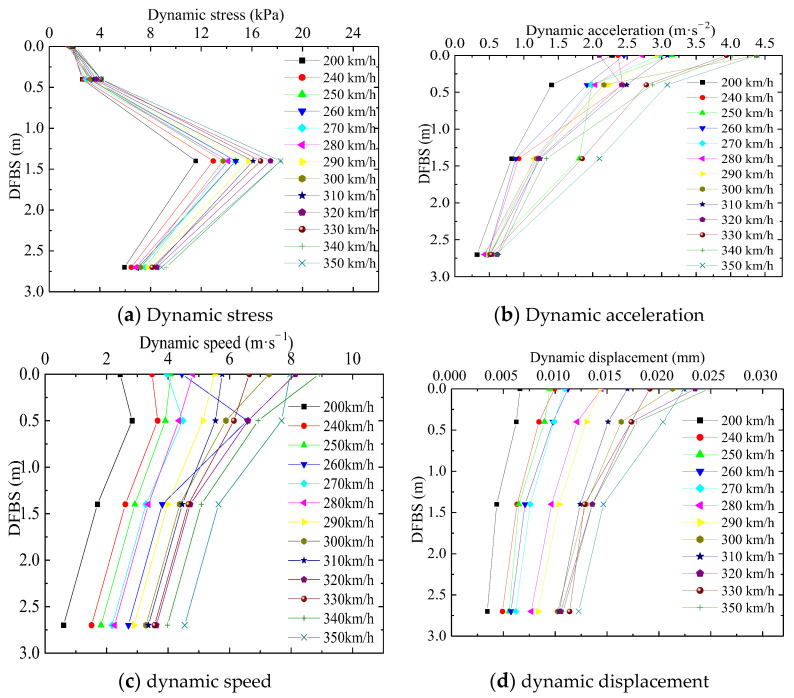
Dynamic response varies with the depth at section DK1252 + 701.075.

Test sensors were embedded at section DK1252 + 701.075 to investigate the ways that dynamic responses change along the subgrade depth, which are shown in Table 5 and Figure 8.

According to Figure 8a,c, the dynamic stress and speed show the same changes along the depth direction. The dynamic speed and dynamic stress first increase along the depth and reach their maximum values when the depth is 1.4 m and 0.5 m, respectively. Then, the dynamic speed and dynamic stress decrease with increasing depth. Most notably, the dynamic stress sharply rises by 859.15% from the top to the bottom of the bed surface and then sharply decreases by 336.52 from the top to the bottom of the sub-base bed. The faster the train runs, the greater the variation is. This may be attributed to the high stiffness of the graded crush stone with 5% cement, which disperses the stress to a wider scope of the subgrade.

Based on Figure 8b,d, as the depth increases, the dynamic acceleration and dynamic displacement decrease until they gradually stabilize. The mean dynamic acceleration and dynamic displacement at the bottom of the bed surface are attenuated by 26.17% and 17.65% from the surface of the bed surface, respectively. The mean dynamic acceleration and dynamic displacement at the bottom of the subgrade are attenuated by 40.40% and 64.69% from the surface of the subgrade, respectively. It should be emphasized that the elastic deformation of the subgrade occurs mainly in the bed layer. It is difficult for an HST to operate at a high speed unless the differential settlement is lower than 2.0 mm, according to *Research on the Design Parameters of Bridges and Tunnels in High-Speed Railways*. The data show that the differential settlement can meet these requirements.

### 5.4. Response to Train Running Speed

**Figure 9:**
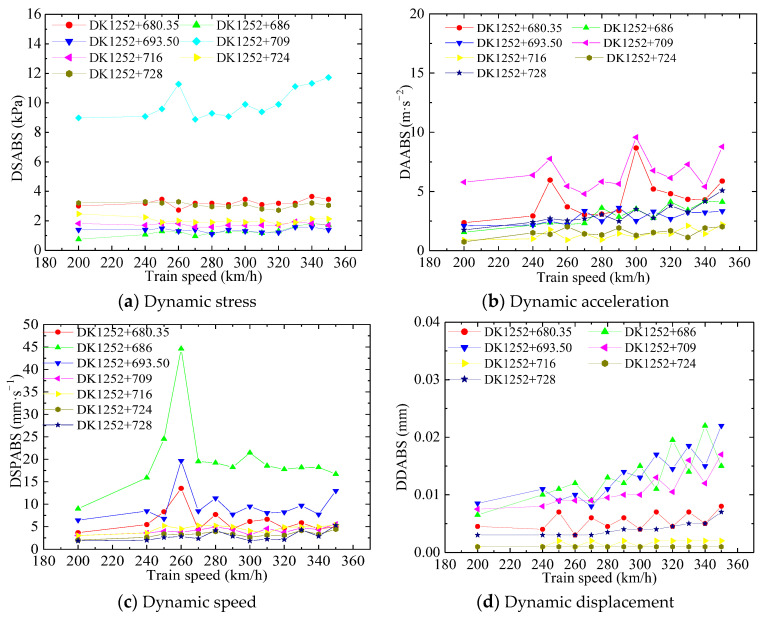
Changes in dynamic response according to train speed on the bed surface.

Figure 9 shows the relationship between the dynamic response and the train running speed. It can be noticed that the dynamic stress changes little as the train running speed increases, indicating that the train running speed does not have much of an effect on dynamic stress. Only the dynamic stress at section DK1252 + 709 is extremely high, which is consistent with our findings that dynamic stress changes with the longitudinal position.

As shown in Figure 9b,c, the changes in the curves of dynamic acceleration and dynamic speed are relatively gentle as the train speed increases. It is obvious that the dynamic response reaches a peak value at 260 km/h and reaches a sub-peak at 300 km/h. Figure 9d shows that dynamic displacement increases gradually as the train speed increases.

To sum up, train speed has a certain (but not significant) effect on the dynamic responses observed at the bed surface. Considering that bed acceleration is the most sensitive representative of the variations in the vibration [50,51], 260 km/h is regarded as a critical train speed.

### 5.5. Response to Train’s Running Direction

**Figure 10:**
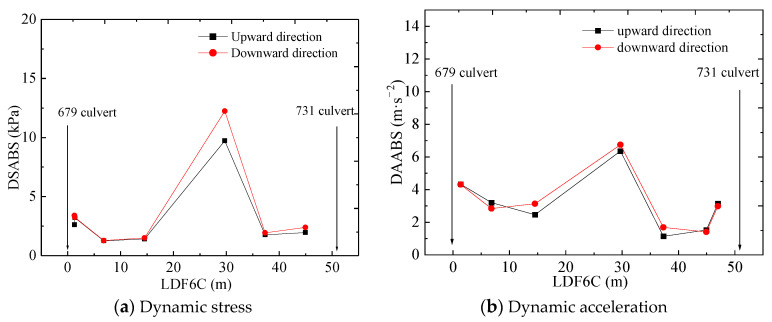

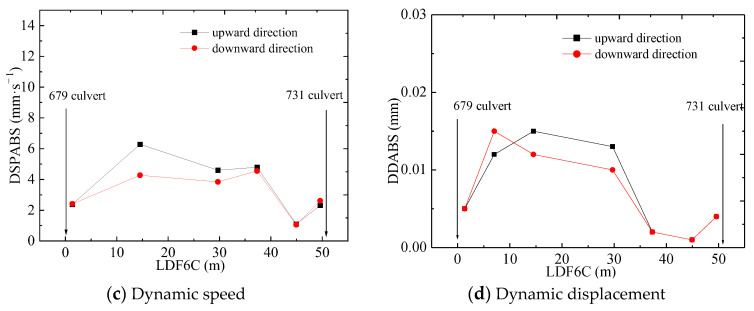
Changes in dynamic response with different running directions.

The changes in the dynamic responses according to the train’s running direction are shown in Table 6 and Figure 10. In terms of dynamic stress, the existence of culverts reduces the stressed area of the culvert bed, causing some stress to concentrate on the two culverts. Another important phenomenon is that the maximum stress differences and maximum acceleration differences appear at section DK1252 + 709 instead of the sections with culverts. When the train runs downward, the stress amplitude increases by 20.59% more than upwards; the acceleration amplitude increases by 32.24%. This infers that the vibration waves caused by the train are superposed to a peak after transferring, reflecting, and refracting in the transition section. Thus, we can conclude that the train’s running direction has an obvious impact on the section between the two culverts.

In terms of the other dynamic responses, they continue to increase as the longitudinal distance from the culvert increases; they reach a peak value at the section between the two culverts and then decrease. Obviously, the train’s running direction shows an influence on the sections between the two culverts, but the influence on the two culverts is not so obvious. When the train runs downward, the speed decreases by 19.79% compared to upwards, and the displacement decreases by 30% compared to upwards, while the dynamic response difference between the train running upward and downward is almost zero. This may be caused by the dynamic waves’ propagation and reflection.

### 5.6. Response to Vehicle Axle Weight

**Figure 11:**
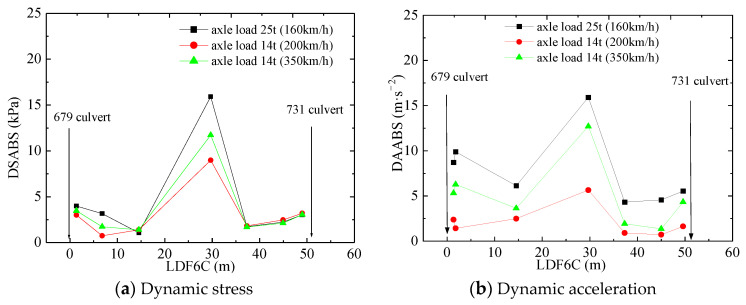

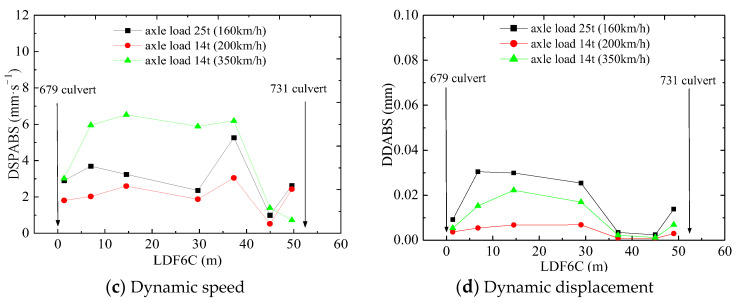
Relationship between dynamic response and axle load.

Figure 11 depicts the relationship between the dynamic responses and the train axle load at train speeds of 160–350 km/h. The dynamic responses at sections DK1252 + 679, DK1252 + 708.7, and DK1252 + 731 are listed in Table 7. The vehicle with an axle load of 25 t in the test is a track inspection train with a running speed of 160 km/h. The vehicle with an axle load of 14 t in the test is an HST with a running speed of 200 km/h or 300 km/h.

Taking section DK1252 + 708.7 as an example, it is clear that the axle load has an impact on the dynamic responses. When the axle load changes from 14 t to 25 t, the stress increases by 77.28% and 35.84% at speeds of 200 km/h and 350 km/h, respectively. Similarly, the acceleration increases by 181.91% and 25.20% at speeds of 200 km/h and 350 km/h, respectively. At the same time, the speed increases by 26.20% and −59.86% at speeds of 200 km/h and 350 km/h, respectively. The dynamic displacement increases by 257.14% and 47.06% at speeds of 200 km/h and 350 km/h, respectively. Furthermore, the dynamic stress and acceleration with a 25 t axle load are greater than with a 14 t axle load, and the dynamic speed and displacement with the 25 t axle load are smaller than those with the 14 t axle load.

The above test data have certain limitations because the train running speeds are different for the two trains, each with different axle loads. However, it is certain that axle load has a great impact on the dynamic responses in the transition section. The faster the train’s speed is, the smaller the impact on dynamic responses is.

## 6. Conclusions

Field tests were carried out to investigate the dynamic characteristics of culvert–embankment–culvert transitions. The test data were processed through denoising, smoothing, and integrating. The main conclusions are as follows:

(1) The graded distribution of graded crushed stone with 5% cement is a suitable subgrade filler according to its physical and mechanical properties, as it is not uniform but is well-graded. Sample #1 is the best roadbed filler for transition sections. Its optimum moisture content is 4.6%, and its unconfined compressive strength after 7 days of curing is 5.5 MPa.

(2) Vibration waves repeatedly reflect and refract due to the complex compositions of the transition section, causing extreme dynamic responses in some special sections. Some special measures should be taken in these sections. In the culvert–embankment–culvert transition areas we studied, the sections 14.5 m and 30 m away from the 679 culvert are the special sections. The bed layer in these areas should be specially consolidated.

(3) Acceleration is the most sensitive variable for transition sections. The top bed is the most sensitive layer to the train load, followed by the bottom bed. Furthermore, 260 km/h is a critical speed for these transition sections.

## Figures and Tables

**Table 1:** Test items and results of raw filling material specifications at the testing site.

Tested Items	Value
Particle size (>22.4 mm) content with crushed surface	45.0%
Wear rate of particles (>1.7 mm)	17.9%
Soaking loss rate of sodium sulfate solution of particles (>1.7 mm)	1.0%
Liquid limit of fine particles (<0.5 mm)	16.8%
Plasticity index of fine particles (<0.5 mm)	3.3%
Content of clay and other impurities	0.9%
Content of needle-shaped soil particles	8.4%

**Table 2:** Grading parameters of three types of graded crushed stone.

Grading Parameters	1#	2#	3#
Cu	28.67	16	16
Cc	1.53	1.56	1.82

**Table 3:** Results of compaction test and test of unconfined compressive strength.

Sample Number	Maximum Dry Density	Optimum Moisture Content	Unconfined Compressive Strength after 7 Days of Curing	Number of Sample Groups
1#	2.36 g/cm^3^	4.6%	5.5 MPa	5
2#	2.32 g/cm^3^	4.2%	3.1 MPa	5
3#	2.35 g/cm^3^	4.5%	5.2 MPa	5

**Table 4:** Comparison of indicators of the proportion of mixed stones for two comparative gradations.

**Virtual laying thickness**	**1#**				
	**K_30_ (MPa/m)**	**E_v2_ (MPa)**	**E_v2_/E_v1_**	**E_vd_ (MPa)**	**Porosity *n* %**
25 cm	260	501.7	2.43	67.3	17.0
30 cm	256	294.3	2.47	84.6	19.6
**Virtual laying thickness**	**2#**				
	**K_30_ (MPa/m)**	**E_v2_ (MPa)**	**E_v2_/E_v1_**	**E_vd_ (MPa)**	**Porosity *n* %**
25 cm	185	365.3	3.76	66.5	21.5
30 cm	173	286.4	4.67	52.5	27.2

Note: K_30_—the ratio of the corresponding load strength p (MPa) and its settlement of 1.25 mm for a load plate with a diameter of 30 cm measured through experiments; E_v1_, E_v2_—primary deformation modulus and secondary deformation modulus for a static modulus. After the first loading and unloading of the ground through a circular bearing plate and loading device, a second loading was carried out. The secant of the slopes between the load settlement curves of 0.3 and 0.7 for the first and second loadings are E_v1_ and E_v2_. The measured stress under the bearing plate and the corresponding settlement at the center of the bearing plate were used. E_vd_—dynamic deformation modulus; the parameter of the soil’s resistance to deformation under a certain vertical impact force F_s_ and impact time t_s_.

**Table 5:** Changes in dynamic responses according to depth in section DK1252 + 701.075.

Dynamic Response	Depth from Top of Bed Surface (m)	Average Value	Percentage of Dynamic Response Decay (%)
Dynamic stress (kPa)	0	1.78	--
	0.4	3.45	−93.82
	1.4	15.57	859.15
	2.7	7.77	−336.52
Dynamic acceleration (m/s^2^)	0	3.21	--
	0.4	2.37	26.17
	1.4	1.34	40.40
	2.7	0.51	84.11
Dynamic speed (mm/s)	0	5.92	--
	0.4	5.57	5.91
	1.4	4.08	64.84
	2.7	2.98	49.66
Dynamic displacement (mm)	0	0.017	--
	0.4	0.014	17.65
	1.4	0.011	64.69
	2.7	0.009	47.06

**Table 6:** Dynamic response amplitudes on the bed surface with different train directions.

Response Type	Distance from 679 Culvert (m)	Upward Direction	Downward Direction	Percentage (%)
Dynamic stress(kPa)	1.27	2.62	3.40	22.94
	6.80	1.26	1.28	1.56
	14.54	1.41	1.50	6.00
	29.70	9.72	12.24	20.59
	37.33	1.76	1.93	8.81
	52	3.02	3.76	19.68
Dynamic acceleration (m/s^2^)	1.27	4.331	4.312	−0.44
	6.80	3.201	2.836	−12.87
	14.54	2.459	3.133	21.51
	29.70	6.356	6.743	5.74
	37.325	1.141	1.684	32.24
	45	1.524	1.404	−8.55
	49.6	3.142	2.985	−5.26
Dynamic speed (mm/s)	1.27	2.243	2.250	0.31
	14.54	6.28	4.27	−47.07
	29.7	4.60	3.84	−19.79
	37.325	4.894	4.779	−2.41
	45	1.256	1.255	−0.08
	49.6	2.32	2.61	11.11
Dynamic displacement (mm)	1.27	0.005	0.005	0.00
	6.80	0.012	0.015	20.00
	14.54	0.015	0.012	−25.00
	29.7	0.013	0.010	−30.00
	37.325	0.002	0.002	0.00
	45	0.001	0.001	0.00
	49.6	0.004	0.004	0.00

**Table 7:** Comparison of dynamic responses with different axle loads.

Dynamic Responses	Section Away from 679 Culvert (m)	HST (Running Speed (km/h))	Track Train (Running Speed (km/h)	Increased Percentage (*%*)
Dynamic stress (kPa)	1.35	3.01 (200)	3.99 (160)	32.56
		3.46 (350)		15.32
	29.7	8.98 (200)	15.92 (160)	77.28
		11.72 (350)		35.84
	49	3.04 (200)	3.03 (160)	-0.33
		3.20 (350)		-5.31
Dynamic acceleration (m/s^2^)	1.35	2.38 (200)	8.71 (160)	265.97
		5.31 (350)		64.03
	29.7	5.64 (200)	15.90 (160)	181.91
		12.70 (350)		25.20
	49	1.63 (200)	5.54(160)	239.88
		4.34 (350)		27.65
Dynamic speed (mm/s)	1.35	1.81 (200)	2.90 (160)	60.22
		3.01 (350)		3.65
	29.7	1.87 (200)	2.36 (160)	26.20
		5.88 (350)		−59.86
	49	2.43 (200)	2.63 (160)	8.23
		0.72 (350)		265.28
Dynamic displacement (mm)	1.35	0.004(200)	0.009 (160)	125.00
		0.005 (350)		80.00
	29.7	0.007(200)	0.025 (160)	257.14
		0.017 (350)		47.06
	49	0.003(200)	0.014 (160)	366.67
		0.007 (350)		100

## Data Availability

The data presented in this study may be available on reasonable request from the first or corresponding author.
